# Pretreatment tumor infiltrating lymphocytes and outcome in patients with HR+/HER2- advanced breast cancer treated with CDK4/6 inhibitors

**DOI:** 10.1038/s41598-026-40616-1

**Published:** 2026-02-26

**Authors:** Rosalba Torrisi, Laura Giordano, Saverio Pancetti, Carlo Carnaghi, Vera Basilico, Raffaella Palumbo, Riccardo Gerosa, Giuseppe Saltalamacchia, Maria Vita Sanò, Armando Santoro, Bethania Fernandes

**Affiliations:** 1 IRCCS, Humanitas Research Hospital Medical Oncology and Hematology Unit, viale Manzoni 56, 20089 Rozzano, MI Italy; 2 Pathology Department Rozzano, IRCCS Humanitas Research Hospital, Milano, Italy; 3 Department of Biomedical Sciences, Humanitas University, Pieve Emanuele, MI Italy; 4 Medical Oncology Unit, Istituto Clinico Humanitas, Centro Catanese di Oncologia, Catania, Italy; 5 Medical Oncology Unit, Istituto Clinico Mater Domini Humanitas, Castellanza, Varese, Italy; 6 Oncologia Medica IRCCS ICS Maugeri, Pavia, Italy

**Keywords:** HR+/HER2 negative advanced breast cancer, CDK4/6 inhibitors, Stromal tumor infiltrating lymphocytes, Biomarkers, Cancer, Immunology, Oncology

## Abstract

Growing evidence suggests that enhancement of the tumor immune response contributes to the antitumor activity of CDK4/6 inhibitors (CDK4/6i), but the role of pretreatment tumor immune microenvironment is not clear. We retrospectively investigated in patients with HR+/HER2- advanced breast cancer receiving CDK4/6i and endocrine therapy whether pretreatment stromal tumor infiltrating lymphocytes (sTILs) were associated with outcome. A total of 100 patients were evaluable 53 treated with palbociclib, 44 with ribociclib and 3 with abemaciclib. Tumors were classified as sTILs positive (sTILs+) when sTILs were ≥ 10%. 58 tumors were sTILs negative (sTILs-) and 42 sTILs+. sTILs were not associated with any outcome overall and in the ribociclib cohort. Conversely, patients treated with palbociclib and sTILs+ tumors experienced a statistically significant improved overall survival (mOS Not Reached vs. 41.1 months, *p* = .038) and a reduced risk of visceral progression (24 vs. 8 patients *p*=.0048) as compared with patients with sTILs- tumors. Pretreatment sTILs levels were associated with outcome in patients treated with palbociclib. Given the lack of interaction between treatment and sTILs our findings warrant validation in larger, independent cohorts and if confirmed propose sTILs as a simple and reproducible biomarker to aid patient selection for palbociclib.

**Supplementary Information:**

The online version contains supplementary material available at 10.1038/s41598-026-40616-1.

## Introduction

The combination of Cyclin-Dependent Kinases 4/6 inhibitors (CDK4/6i) and endocrine therapy (ET) represents the standard treatment for HR+HER2- advanced breast cancer (ABC)^1^.

Studies in preclinical models and in patients indicate that, in addition to a direct effect on the tumor, CDK4/6i might influence the tumor immune microenvironment providing a strong rationale for combining CDK4/6i and immunotherapy^2–4^. Pharmacologic inhibition of CDK4/6 increased levels of tumor-infiltrating T cells in vivo, and synergized with anti–PD-1 blocking antibodies in multiple syngeneic tumor models^2,4^.

In breast tumor tissues of patients treated with abemaciclib regulation of tumor–secreted cytokines and upregulation of PD-L1 expression, a significant reduction of CD4 + FOXP3+ TRegs but not of CD8 + T cells have been observed^3,5^. The selective suppression of TReg proliferation (and not that of CD8 + or conventional CD4 + T cells) has been related to the higher levels in TReg cells of *Rb1*, a key mediator of CDK4/6 pathway modulation and highly retained in HR+ tumors^4^.

However, the role of pretreatment tumor immune microenvironment is not clear.

Stromal tumor-infiltrating lymphocytes (sTILs) are the most reproducible immune parameter scored by pathologists^6^. Conflicting results have been reported on the role of preatreatment sTILs in predicting resistance or benefit from treatment with CDK4/6i and ET^7–9^.

The Solti Group described a statistically significant negative association between higher sTILs and progression-free survival (PFS) while Schettini et al. showed a favorable survival trend in patients with higher sTILs^7,8^. However, the number of sTILs positive (TILs+) tumors in both studies (19 and 16) make these findings exploratory. We have previously shown a not statistically significant improved PFS for patients treated with palbociclib and pretreatment sTILs+ tumors, but the small proportion of sTILs+ tumors prompted the need to extend this investigation in larger samples^9^.

In this study we examined a larger cohort of women with HR+/HER2- ABC treated with CDK4/6i and ET and correlated the presence of sTILs with clinical and pathological features, patterns of progression and outcomes.

## Patients and methods

We retrospectively investigated whether sTILs in tissue biopsies of a consecutive series of patients with HR+/HER2- ABC obtained before treatment with a CDK4/6i and ET were associated with pathological and clinical features, patterns of progression and clinical outcomes.

Pre- and postmenopausal women and males treated up to the 3rd line with a CDK4/6i and ET (aromatase inhibitors or Fulvestrant + GnRH-analogue if premenopausal or males) for ≥ 12 months or progressing earlier were eligible. Patients had sTILs measured in tissues from metastatic sites (or from breast tumor if synchronous metastases not accessible) obtained very close to treatment start with CDK4/6i (at maximum 3 months) with no intermediate treatment.

Information on age and menopausal status at treatment, start, previous therapies, pathological features of metastatic tumor, sites of metastatic disease at treatment start and at progression, last follow up visit and death if occurred were collected.

sTILs were assessed and scored in FFPE tumor biopsies at the Pathology Department of Humanitas Research Hospital according to the recommendations of the TILs working group by two experienced pathologists who were blinded to patient clinical data^6^. In particular, sTILs were reported for the stromal compartment (=% stromal TILs). sTILs were evaluated within the borders of the invasive tumor. All mononuclear cells (including lymphocytes and plasma cells) were scored, except of polymorphonuclear leukocytes. One Sects. (4–5 μm, magnification ×200–400) per patient was considered to be sufficient (6). Tumors were classified as sTILs + when the stromal infiltration was ≥ 10%. sTILs values were rounded to nearest 5%.There is no consistent agreement for the choice of a definite a cut-off particularly in HR+/HER2- breast cancer^10^ but according to literature recommendations, as well as to retrospective and prospective studies which have considered sTILs+ tumors when sTILs were ≥ 10%^11–13^, we chose the 10% cut-off which according to the observed sTIls distribution it seemed to better discriminate between negative and positive tumors in our cohort (Supplementary Table 1).

The study was approved by the Institutional Ethical Committee of Humanitas Research Hospital (n 22/25, 22.04.25). The study complied with all relevant ethical regulations regarding patient data, in line with ethical norms and standards in the Declaration of Helsinki. All patients gave their informed consent.

### Statistical analyses

Categorical variables were summarized using absolute frequencies and proportions, while continuous variables were described by median and range. Group comparisons for categorical data were performed using the Chi-square test or Fisher’s exact test, as appropriate. For continuous variables, the t-test was used when data followed a normal distribution, and the Wilcoxon Mann–Whitney U test was applied for non-normally distributed data.

The primary outcome measures were Progression-Free Survival (PFS) and Overall Survival (OS), both calculated from the date of the first dose of CDK4/6i. Kaplan–Meier methods were used to estimate survival curves for PFS and OS, and group differences were assessed using the log-rank test. Follow-up was estimated using the reverse Kaplan–Meier method^14^.

To control for potential confounding factors and provide adjusted estimates, multivariable analyses were conducted using the Cox proportional hazards model. Hazard ratios (HRs) and corresponding 95% confidence intervals (CIs) were calculated. The proportional hazards assumption was formally tested and satisfied. In addition, potential interactions between key covariates (e.g., treatment cohort, HER2 status defined as HER2 0 vs. HER2 low which included immunohistochemical scoring 1 + and 2+/ISH negative, and sTILs) were explored.

For the analysis of 24-month PFS, probabilities were derived directly from Kaplan–Meier estimates, and odds ratios (OR) with 95% confidence intervals were calculated from these estimates, properly accounting for censored data.

A p-value < 0.05 was considered statistically significant in all analyses. All statistical computations were performed using SAS software, version 9.4.

## Results

A total of 108 patients were evaluated for the study: 50 patients derived from the previous study^9^ and 58 additional consecutive patients treated with ET and CDK4/6i at the Humanitas Research Hospital for whom TILs assessment in pretreatment biopsies were available. 100 patients fulfilled eligibility criteria and were included in the analysis after excluding 4 patients from the 1st cohort (3 who had stopped treatment > 12 months before progression or last follow up and 1 patient who had received ET+CDK4/6i in 6th line) and 4 patients from the 2nd cohort (3 who did not have valuable disease and 1 patient who had stopped treatment before 12 months without PD and did not have further follow up).

Baseline patient characteristics are summarized in Table 1.

**Table 1:** Patient characteristics.

	OVERALL
***N*** * patients*	100
**Age** years mean (range)	59.5 (20–85)
**MBC**	
De novo	38
Recurrent	62
**Menopausal status**	
Pre-perimenopausal	33
Postmenopausal	65
Not applicable (male patients)	2
**HER2 status**	
HER2 0	46
HER2 low	54
**Tumor subtype**	
Luminal A-like	51
Luminal B-like	41
Not classified	8
**Previous adjuvant therapy**	
chemotherapy	34
Endocrine therapy	57
**Metastatic disease**	
Not visceral	50
Visceral	50
**Metastatic sites**	
< 3	71
≥ 3	29
**Biopsy**	
Breast	34
Visceral	34
Soft tissues	32
**CDK 4/6i line of therapy**	
1st	86
>2nd	14
**CDK 4/6 i**	
Abemaciclib	3
Palbociclib	53
Ribociclib	44
**Endocrine agent**	
Aromatase inhibitors	72
Fulvestrant	28
**ECOG PS**	
0	69
≥1	31

MBC metastatic breast cancer; CDK4/6i Cyclin-Dependent Kinases 4/6 inhibitors; PS Performance status.

58 tumors were sTILs negative (sTILs-) and 42 were sTILs+. sTILs were not statistically significantly associated with any clinical and pathological feature (Table 2).

**Table 2:** Pathological and clinical characteristics according to stromal Tumor infiltrating lymphocyte (sTILs) status.

	sTILs negative *N* (%)	sTILs positive *N* (%)	*p*-value
***N*** ** patients**	58	42	
**Age**,** years mean (range)**	60.5 (20–85)	56.5 (29–83)	0.08
**MBC**			0.66
De novo	21 (55.3)	17 (44.7)	
Recurrent	37 (59.7)	25 (40.3)	
**Menopausal status**			0.07
Pre-/perimenopausal	15 (45.5)	18 (54.5)	
Postmenopausal	42 (64.6)	23 (35.4)	
**HER2 status**			0.08
HER2 0	31 (67.4)	15 (32.6)	
HER2 low	27 (50.0)	27 (50.0)	
**Tumor subtype**			0.38
Luminal A	32 (62.7)	19 (37.3)	
Luminal B	22 (53.7)	19 (46.3)	
**Metastatic disease**			0.69
Not visceral	30 (60.0)	20 (40.0)	
Visceral	28 (56.0)	22 (44.0)	
**Metastatic sites**			0.94
< 3	41 (57.7)	30 (42.3)	
≥ 3	17 (58.6)	12 (41.4)	
**Biopsy site**			0.86
Breast	19 (55.9)	15 (44.1)	
Visceral	18 (56.3)	14 (43.8)	
Soft tissue	21 (61.8)	13 (38.2)	
**CDK4/6i line of therapy**			0.09
1st	47 (54.7)	39 (45.3)	
> 2nd	11 (78.6)	3 (21.4)	
**CDK4/6 i**			0.58
Palbociclib	33 (62.3)	20 (37.7)	
Ribociclib	23 (52.3)	21 (47.7)	
Abemaciclib	2 (66.7)	1 (33.3)	
**Endocrine agent**			0.21
Aromatase inhibitors	39 (54.2)	33 (45.8)	
Fulvestrant	19 (67.9)	9 (32.1)	

MBC metastatic breast cancer; CDK4/6i Cyclin-Dependent Kinases 4/6 inhibitors.

At a median follow-up of 55.6 months (range 4.4–89.5) mPFS was 21.1 months and 30.2 months in patients with sTILs- and sTILs+ tumors, respectively (*p* =.2,). The distribution of TILs+ tumors was similar across the CDK4/6i (Table 2).

Patients with sTILs- tumors treated with palbociclib had a significantly worse PFS as compared with those treated with ribociclib (*p*=.011), while no difference was observed in patients with sTILs+ tumors (*p* =.64).

**Fig. 1:**
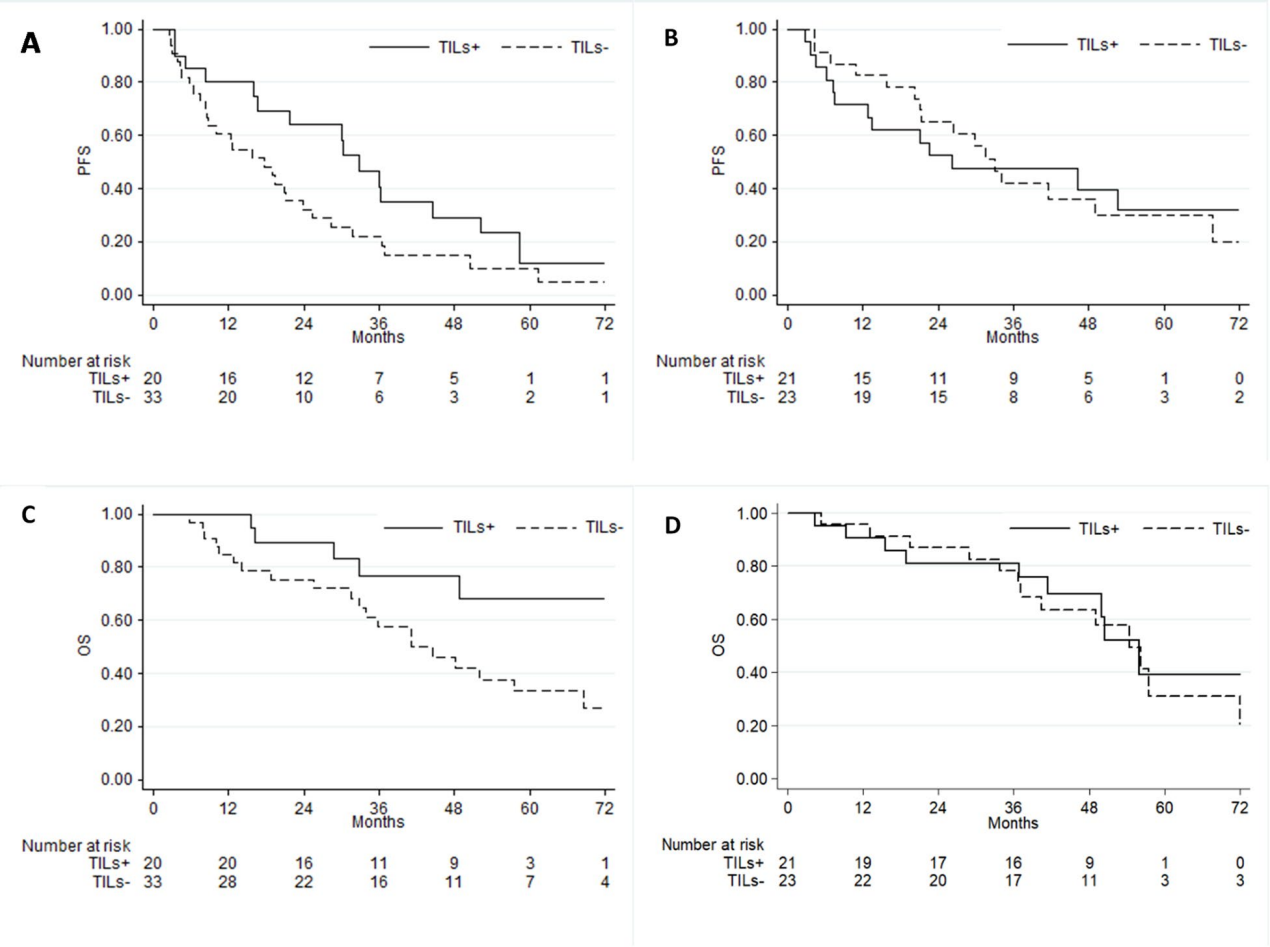
Upper panel: progression -free survival according to TILs in (**A**) patients treated with palbociclib; (**B**) patients treated with ribociclib. Lower panel: Overall survival according to TILs in (**C**) patients treated with palbociclib; (**D**) patients treated with ribociclib. PFS: progression-free survival; TILs: Tumor infiltrating lymphocytes; OS; overall survival.

Among patients treated with palbociclib there was a trend for a longer PFS in patients with sTILs+ tumors (mPFS 32.7 vs. 17.6 months in sTILs– tumors (*p*=.06), while in patients treated with ribociclib no difference was observed: mPFS = 33 and 26.3 months in sTILs- and + tumors, respectively (*p* =.99). (Table 3; Fig. 1)

**Table 3:** Outcomes according to tumor infiltrating lymphocytes status and CDK4/6 inhibitor.

	OVERALL	*p*-value	PALBOCICLIB *N* = 53	*p*-value	RIBOCICLIB *N* = 44	*p*- value
mPFS months sTILs– sTILs+	21.1 (15.8–31.5) 30.2 (16- 46.2)	0.21	17.6 (8.5- 23-8) 32.7 (16- 52.2)	0.06*	33 (21–67.7.7) 26 − 3 (7.5-NR)	0.99
mOS months sTILs- sTILs+	49.1 (36.7–57.4) NR (48.8-NR)	0.11	41.1(31.5–68.6) NR (32.8-NR)	0.038	54.4 (36.7–79.3) 55.9 (41.5-NR)	0.84

CDK4/6i cyclin-dependent kinase 4/6 inhibitors; mPFS median progression-free survival; mOS median overall survival; sTILs- stromal tumor infiltrating lymphocytes negative; sTILs+ stromal tumor infiltrating lymphocytes positive; NR not reached.

The association between sTILs, modeled as a continuous variable, and PFS was evaluated, with no statistically significant relationship observed in the total cohort or within treatment subgroups (Supplementary Table 2).

In multivariable analysis of PFS, including variables which showed a borderline association with sTILs in Table 2, only line of treatment was statistically significantly associated with PFS in the palbociclib cohort (HR = 3.67, *p*=.002) while sTILs showed a favorable (HR = 0.58, *p*=.120) but not statistically significant effect in the same group. No effect was observed with ribociclib (HR = 0.98, *p* =.955).

At 24 months, the probability of remaining progression-free was 64.0% in patients with sTILs+ tumors and 32.1% in patients with sTILs− tumors treated with palbociclib. The corresponding odds ratio calculated from Kaplan–Meier estimates to account for censored observations, for PFS ≥ 24 months was 4.27 (95% CI 1.31–13.9), indicating a statistically significant higher likelihood of prolonged PFS in patients with sTILs+ tumors. For patients treated with ribociclib, the probability of remaining progression-free at 24 months was 52.4% for sTILs + and 65.2% for sTILs− tumors. The corresponding odds ratio, was 0.59 (95% CI 0.18–1.97), indicating no statistically significant difference in 24-month PFS between sTILs + and sTILs− tumors.

Among 50 patients developing visceral progression, 33 (66%) had sTILs- and 17 (34%) sTILs+ tumors (*p* =.1). Patients with TILs- tumors treated with palbociclib had a higher risk of developing visceral progression (24 vs. 8 patients, *p*=.0048). Among patients treated with ribociclib only 18 experienced visceral PD with no difference according to sTILs (*p* =.82).

In patients progressing at other sites no association with sTILs status overall and in the 2 groups was observed.

Median OS was 49.1 months (36.7–57.4) in patients with sTILs- and Not Reached (48.8-NR) in patients with sTILs+ tumors (*p* =.1). Among patients treated with palbociclib patients with sTILs+ tumors experienced a statistically significant improved OS as compared with those with sTILs- tumors (mOS Not Reached (32.8-NR) vs. 41.1 months (31.5–68.6) *p*=.038) (Table 3 Fig. 1). In patients treated with ribociclib mOS was 54.4 (36.7–79.3) and 55.9 months (41.5-NR) in patients with sTILs- and sTILS+ tumors, respectively (*p* =.84) (Table 3; Fig. 1).When sTIL status was evaluated as a continuous variable no statistically significant association with OS was observed overall and in the ribociclib cohort but a trend towards a favourable effect of sTILs in patienst treated with palbociclib was maintained (supplementary Table 2).

In multivariable analysis in the palbociclib-treated cohort sTILs showed a favorable but non-significant association with OS (HR 0.54, *p*=.226), whereas visceral progression was significantly associated with worse OS (HR 5.3, *p*=.008). In the ribociclib cohort no effect was observed for sTILs (HR = 1.11, *p*=.82) while visceral progression was the only statistically significant factor (HR = 6.98, *p*<.001).

No other statistically significant association was observed considering all other possible confounders. The interaction test between treatment and sTILs was not statistically significant (0.154, and 0.130 for PFS and OS respectively).

## Discussion

Traditionally, HR+/HER2- breast cancer has been considered a “cold”, not highly immunogenic tumor, being characterized by a lower tumor mutation burden (TMB), lower rate of PD-L1 expression and of TILs as compared to other breast tumor subtypes^15^.

Growing evidence suggests that the interplay with the immune microenvironment contributes to the antitumor activity of CDK4/6i, the large part of these data pointing to an enhancement of antitumor immune response^2–4,16^. In the RIBECCA study treatment with ribociclib led to an upregulation of signatures associated with an activated adaptive immune system and a decrease in immunosuppressive cytokine signaling in patients with HR+ breast cancer^17^. These findings have provided a rationale for combining CDK4/6i and immune checkpoint inhibitors with encouraging efficacy results despite some safety alerts^18^. On the other hand, the status of the pretreatment tumor immune microenvironment has been less studied with contradictory results^7–9^.

Our findings consistently suggest that pretreatment sTILs measured with a cut-off of 10% are positively associated with outcomes in patients treated with palbociclib and ET. Patients with sTILs+ tumors had a statistically significant longer OS as compared with those with sTILs- tumors. Moreover, in the palbociclib cohort, patients with sTILs+ tumors had a higher probability of PFS ≥ 24 months and a statistically significantly lower risk of experiencing a visceral progression which might led to the improved OS. Notably, no difference between palbociclib and ribociclib was observed in patients with sTILs+ tumors while patients with sTILs- tumors treated with the former drug experienced a significantly worse PFS as compared with those treated with ribociclib.

Interestingly, no association between sTILs status and ribociclib was observed.

To our knowledge, this is the first study suggesting a positive association between pretreatment tumor sTILs, assessed by routine immunohistochemical workup, and outcome on treatment with different CDK4/6i and ET in HR+/HER2- ABC.

Single-cell RNA sequencing of tumor tissues collected in patients treated with CDK4/6i (mostly palbociclib) and ET showed higher levels of CD8 + cytotoxic T cells and NK cells in responders as compared with those of patients with early progression, suggesting that these cells are potential predictive biomarkers for CDK4/6i response^19^.

On the contrary, the NeoPalAna study showed a negative association between baseline sTILs and response to neoadjuvant palbociclib and anastrozole^20^. No association between sTILs status and response to CDK4/6i (mostly palbociclib in 93.7%) and ET was shown in 222 patients with HR+/HER2- ABC^21^.

Similarly, divergent results arise from 2 neoadjuvant studies with ribociclib: the FELINE study showed some differences in pretreatment tumor microenvironment between responders vs. resistant patients^22^, while the CORALLEEN trial failed to show a correlation between pretreatment sTILs and those assessed in 14-days and surgical specimens^23^.

It is unclear how to interpreter these discrepancies among studies and with our findings. To explain the inconsistent results obtained with palbociclib we may hypothesize that tumor immune microenvironment differs between early and advanced tumor; on the other hand, we cannot rule out that distinct components of the immune tumor microenvironment rather than sTILs themselves might be associated with ribociclib sensitivity.

Some differences in the interplay between the host immune system and CDK4/6i has been suggested also in a previous study investigating the behavior of autoimmune diseases in patients treated with different CDK4/6i^24^. Specifically, new and/or flaring autoimmune events and CDK4/6i were observed mostly during treatment with abemaciclib and ribociclib but not with palbociclib although the small number of events prevented a statistical comparison^24^.

The main limitation of our study is the lack of a translational analysis providing a biological rationale underlying the different sensitivity to baseline immune microenvironment of the two CDK4/6i. Another limitation is the limited sample size which requires necessarily validation in larger cohorts. Moreover, the retrospective design did not allow consistent tumor assessment, despite the vast majority of patients were treated at the same Institution following internal guidelines. Strength of the study is sTILs assessment in pretreatment biopsies, which was performed all at the same Pathology Department as was most (87%) of the pathological analyses. In addition, sTILs status was assessed by routine immunohistochemical workup which is feasible in all Pathology Departments.

In conclusion, our results suggest that pretreatment sTILs are associated with outcomes in patients with ABC treated with palbociclib but not with ribociclib. Given the lack of an interaction between sTILs and treatment, and the limited sample size these findings should be considered exploratory and warrant confirmation in larger, prospective studies with dedicated translational studies. Nevertheless, if confirmed, sTILs might represent an easily assessable and reproducible biomarker to aid patient selection for palbociclib.

## Supplementary Information

Below is the link to the electronic supplementary material.

Supplementary Material 1

## Data Availability

The datasets generated and/or analyzed during the current study are available in the repository Zenodo [https://zenodo.org/records/10055012].
