# Supplementary material for: Pretreatment tumor infiltrating lymphocytes and outcome in patients with HR+/HER2- advanced breast cancer treated with CDK4/6 inhibitors
Source: Sci Rep. 2026 Feb 26;16:11161. doi: 10.1038/s41598-026-40616-1 (PMC13047036; doi:10.1038/s41598-026-40616-1)
Supplement: Supplementary file 1 — Supplementary Material 1 [file 41598_2026_40616_MOESM1_ESM.docx]

**PRETREATMENT tumor infiltrating lymphocytes and outcome in patients with HR+/HER2- advanced breast cancer treated with CDK4/6 inhibitors**

SHORT TITLE: **TUMOR INFILTRATING LYMPHOCYTES AND OUTCOME WITH CDK 4/6 INHIBITORS**

Rosalba Torrisi ^1*^, Laura Giordano ^1^, Saverio Pancetti ^2,3^, Carlo Carnaghi ^4^, Vera Basilico ^5^, Raffaella Palumbo^6^, Riccardo Gerosa ^1,3^, Giuseppe Saltalamacchia ^1^ ,Maria Vita Sanò ^4^, Armando Santoro ^1,3^, Bethania Fernandes ^2^

1. IRCCS, Humanitas Research Hospital Medical Oncology and Hematology Unit, viale Manzoni 56 20089 Rozzano (MI, Italy
2. IRCCS Humanitas Research Hospital, Pathology Department Rozzano, Milano- Italy
3. Department of Biomedical Sciences, Humanitas University, Pieve Emanuele (MI), Italy
4. Medical Oncology Unit, Istituto Clinico Humanitas, Centro Catanese di Oncologia, Catania, Italy
5. Medical Oncology Unit, Istituto Clinico Mater Domini Humanitas, Castellanza, Varese- Italy
6. Oncologia Medica  IRCCS ICS Maugeri, Pavia Italy

*Corresponding Author Rosalba Torrisi, Humanitas Research Hospital IRCCS, Medical Oncology and Hematology Unit, viale Manzoni 56 20089 Rozzano (MI), Italy [roslaba.torrisi@gmail.com](mailto:roslaba.torrisi@gmail.com), +39 0282245918.

Supplementary Table 1 Tumor infiltrating lymphocytes distribution

| **sTILs** | **Frequency** | **%** |
| --- | --- | --- |
| **0** | 53 | 53 |
| **5** | 5 | 5 |
| **10** | 25 | 25 |
| **15** | 5 | 5 |
| **20** | 8 | 8 |
| **30** | 4 | 4 |

sTILs stromal tumor infiltrating lymphocytes

Supplementary Table 2 . Outcomes according to stromal tumor-infiltrating lymphocytes (sTILs) status (categorical and continuous) and CDK4/6 inhibitor

|  | **OVERALL** | **p-value** | **PALBOCICLIB** | **p-value** | **RIBOCICLIB** | **p-value** |
| --- | --- | --- | --- | --- | --- | --- |
| **Categorical analysis** |  |  |  |  |  |  |
| mPFS, months |  |  |  |  |  |  |
| sTILs − | 21.1 (15.8–31.5) |  | 17.6 (8.5–23.8) |  | 33.0 (21–67.7) |  |
| sTILs + | 30.2 (16.0–46.2) | 0.21 | 32.7 (16.0–52.2) | 0.06 | 26.3 (7.5–NR) | 0.99 |
| **mOS, months** |  |  |  |  |  |  |
| sTILs − | 49.1 (36.7–57.4) |  | 41.1 (31.5–68.6) |  | 54.4 (36.7–79.3) |  |
| sTILs + | NR (48.8–NR) | 0.11 | NR (32.8–NR) | 0.038 | 55.9 (41.5–NR) | 0.84 |
| **Continuous analysis** |  |  |  |  |  |  |
| **PFS, HR (95% CI)** | 0.99 (0.96–1.02) | 0.51 | 0.98 (0.94–1.01) | 0.199 | 1.02 (0.96–1.08) | 0.535 |
| **OS, HR (95% CI)** | 0.97 (0.93–1.01) | 0.18 | 0.95 (0.89–1.001) | 0.082 | 1.01 (0.95–1.09) | 0.717 |

sTILs, stromal tumor-infiltrating lymphocytes; CDK4/6i, cyclin-dependent kinase 4/6 inhibitors; mPFS, median progression-free survival; mOS, median overall survival; HR, hazard ratio; CI, confidence interval; NR, not reached.
